# A study on the dissolution rates of K-Cr(VI)-jarosites: kinetic analysis and implications

**DOI:** 10.1186/s12932-016-0035-7

**Published:** 2016-06-13

**Authors:** Iván A. Reyes, Ister Mireles, Francisco Patiño, Thangarasu Pandiyan, Mizraim U. Flores, Elia G. Palacios, Emmanuel J. Gutiérrez, Martín Reyes

**Affiliations:** Instituto de Metalurgia, Universidad Autónoma de San Luis Potosí, Av. Sierra Leona 550, Lomas 2a Sección, C.P. 78210 San Luis Potosí, S.L.P. México; Área Académica de Ciencias de la Tierra y Materiales, Universidad Autónoma del Estado de Hidalgo, Carretera Pachuca–Tulancingo km 4.5, C.P. 42081 Mineral de la Reforma, Hgo. México; Ingeniería en Energía, Universidad Politécnica Metropolitana de Hidalgo, Boulevard acceso a Tolcayuca 1009, Ex-Hacienda San Javier, C.P. 43860 Tolcayuca, Hgo. México; Facultad de Química, Universidad Nacional Autónoma de México, Ciudad Universitaria, C.P. 04510 Mexico, D.F. México; Área de Electromecánica Industrial, Universidad Tecnológica de Tulancingo, Camino a Ahuehuetitla 301, Las Presas, C.P. 43642 Tulancingo, Hgo. México; Unidad Profesional Adolfo López Mateos, Escuela Superior de Ingeniería Química e Industrias Extractivas, Instituto Politécnico Nacional, C.P. 07738 Mexico, D.F. Mexico

**Keywords:** Jarosite-type compounds, Chromate analog, Dissolution rates, Kinetic analysis, Stability

## Abstract

**Background:**

The presence of natural and industrial jarosite type-compounds in the environment could have important implications in the mobility of potentially toxic elements such as lead, mercury, arsenic, chromium, among others. Understanding the dissolution reactions of jarosite-type compounds is notably important for an environmental assessment (for water and soil), since some of these elements could either return to the environment or work as temporary deposits of these species, thus would reduce their immediate environmental impact.

**Results:**

This work reports the effects of temperature, pH, particle diameter and Cr(VI) content on the initial dissolution rates of K-Cr(VI)-jarosites (KFe_3_[(SO_4_)_2 − X_(CrO_4_)_X_](OH)_6_). Temperature (T) was the variable with the strongest effect, followed by pH in acid/alkaline medium (H_3_O^+^/OH^−^). It was found that the substitution of CrO_4_^2−^in *Y*-site and the substitution of H_3_O^+^ in *M*-site do not modify the dissolution rates. The model that describes the dissolution process is the unreacted core kinetic model, with the chemical reaction on the unreacted core surface. The dissolution in acid medium was congruent, while in alkaline media was incongruent. In both reaction media, there is a release of K^+^, SO_4_^2−^ and CrO_4_^2−^ from the KFe_3_[(SO_4_)_2 − X_(CrO_4_)_X_](OH)_6_ structure, although the latter is rapidly absorbed by the solid residues of Fe(OH)_3_ in alkaline medium dissolutions. The dissolution of KFe_3_[(SO_4_)_2 − X_(CrO_4_)_X_](OH)_6_ exhibited good stability in a wide range of pH and T conditions corresponding to the calculated parameters of reaction order *n*, activation energy *E*_*A*_ and dissolution rate constants for each kinetic stages of induction and progressive conversion.

**Conclusions:**

The kinetic analysis related to the reaction orders and calculated activation energies confirmed that extreme pH and T conditions are necessary to obtain considerably high dissolution rates. Extreme pH conditions (acidic or alkaline) cause the preferential release of K^+^, SO_4_^2−^ and CrO_4_^2−^ from the KFe_3_[(SO_4_)_2 − X_(CrO_4_)_X_](OH)_6_ structure, although CrO_4_^2−^ is quickly adsorbed by Fe(OH)_3_ solid residues. The precipitation of phases such as KFe_3_[(SO_4_)_2 − X_(CrO_4_)_X_](OH)_6_, and the absorption of Cr(VI) after dissolution can play an important role as retention mechanisms of Cr(VI) in nature.

## Background

Chromium (Cr) is one of the most strategic materials in the world. Due to its toxic nature, Cr poses several environmental problems, namely waste products, such as mining waste and battery slag disposed of after manufacturing. Waste products, usually contain Cr(VI) as chromic acid, but also a low amount of reduced Cr(III) and Cr as solid metal [1]. High Cr content solutions released in soils by leakage or inadequate waste disposal by industrial facilities can alter the chemical environment of soils. This can result in the dissolution of minerals native to the soil, or in the precipitation of new phases that have the capability to incorporate high concentrations of Cr. These precipitates can limit the mobility of Cr(VI) and therefore, its bioavailability, like the Cr phase identified by Baron et al. [2] in soil polluted by chromate solutions. These phase was identified as KFe_3_(CrO_4_)(OH)_6_, which is the structural analog of jarosite KFe_3_(SO_4_)(OH)_6_. Sulfate is a natural component of soils and underground waters, and it is present in chromate solutions, which are one of the main causes of pollution by Cr. Sulfate and chromate have the same equivalent charge (2−), same crystal structure and similar ionic radii (2.30 Å for SO_4_^2−^ and 2.40 Å for CrO_4_^2−^). Additionally, an extensive literature search related to the formation of solid solutions in the alunite/jarosite group suggests the existence of the solid solution KFe_3_[(SO_4_)_2 − X_(CrO_4_)_X_](OH)_6_ between jarosite and its chromate analog [3, 4]. The presence of these solid solutions could have important implications in the mobility of Cr(VI). Understanding the dissolution reactions of these solid solutions is notably important for an environmental assessment of the effects of chromium, because Cr(VI) frequently enters the environment [5].

Jarosite, its chromate analog and its solid solutions belong to the alunite supergroup, whose general formula is MY_3_(ZO_4_)_2_(OH)_6_, where *M* = Na^+^, K^+^, Ag^+^, Rb^+^, H_3_O^+^, Tl^+^, NH_4_^+^, ½ Hg^2+^, ½ Pb^2+^; *Y* = Fe^3+^, Al^3+^, Cr^3+^, Cu^2+^, Zn^2+^; Z = S(VI), Cr(VI), As(V) or P(V). The alunite supergroup is composed by three mineral groups: alunite group, where ZO_4_ is represented by SO_4_ as dominant anion in the minerals; beudantite group, where one of the two SO_4_ groups is replaced by PO_4_ or AsO_4_; crandallite group, where ZO_4_ is represented by one or both PO_4_ and AsO_4_. The combination of these groups can form more than 40 different compounds [6]. Particularly in jarosite-type compounds, *Y*-site is occupied by Fe^3+^, and *Z*-site is occupied by S(VI). Although nine jarosite compounds can be synthesized, only six of them have been found in nature as minerals, the most common being sodium, potassium and hydronium jarosite. Silver, ammonium and lead jarosite have been also found in nature [7]. Rubidium, thallium and mercury jarosites are considered pure phases, because they can completely substitute *M*-site, even if they can only be obtained by synthetic means [8, 9]. Substitution in *M*-site of hydronium ions by potassium or sodium ions shows that most of the jarosite-type compounds are solid solutions of hydronium jarosite [10, 11]. Partial substitutions by Cs^+^ and ½ Cd^2+^, and null substitution of Li^+^ on *M*-site have been reported by Dutrizac and Jambor [12], and Dutrizac [13]. Besides the substitutions on site *M*-site, it is well established that several species can substitute Fe, SO_4_, and at a lesser extent, OH, which are structural components of jarosite-type compounds [14]. Complete substitutions of Al(III), In(III), Ga(III) and Cr(III) on *Y*-site as well as a partial substitution by Tl(III) have been reported. In contrast, substitutions by Y(III), Sc(III), U(III) and others rare earths do not occur. Complete and partial substitutions of SO_4_^2−^ on *Z*-site by SeO_4_^2−^, CrO_4_^2−^ and AsO_4_^3−^, and partial substitutions of F^−^ by OH^−^ have been observed in synthetic and natural jarosites [3, 7, 8, 10, 12], [14–23]. As it can be noticed, jarosite-type compounds can undergo several kinds of substitutions thanks to the different coordination environments in its structure. Some of those substitutions can be made by elements of environmental importance, such as Tl^+^, ½ Pb^2+^, ½ Hg^2+^, ½ Cd^2+^, Tl^3+^, Cr(VI), As(V), and these compounds can work as temporary deposits of these species, thus reducing their environmental impact. Jarosite-type compounds are naturally formed under acidic conditions, during oxidation of sulfurous mineral deposits or ores that contain sulfide, namely pyrite [24]. This is mostly due to supergenic and hydrothermal processes [25]. However, they are also commonly found in places polluted by acid rock drainage (ARD) and acid mine drainage (AMD). The precipitation of jarosites is also used in the hydrometallurgical industry in the elimination of Fe and other impurities in acid solutions from leaching processes of zinc sulfate, copper sulfate and cobalt sulfide [26].

Several studies have been conducted in order to understand the precipitation/dissolution process of natural and synthetic jarosite-type compounds [3, 4, 20], [27–29]. These works have been focused mainly on studying the effects of pH on the dissolution of K, Pb, Pb-As and K-Cr jarosites using different reaction media. It is worth mentioning that these research studies were conducted in the steady state of the dissolution reaction (i.e. in equilibrium). Likewise, research has been developed focusing on the initial states of the reaction of K, Na, K-As and Na-As jarosites, where the highest release of species into the solution has been reported [30–35]. In addition, research studies have been conducted in order to know the thermodynamic properties, [11], [36–38] and dissolution for the recovery of metal values, like Ag, from Ag, Pb-Ag, Na-Ag, K-Ag, NH_4_-Ag and industrial NH_4_ jarosites [39–45]. The aim of this paper is to present a detailed kinetic study on the dissolution of potassium jarosite, its chromate analog, and its solid solutions under extreme temperature and pH conditions, in order to obtain information for the assessment on the potential environmental impact of Cr(VI) in the initial stage of the dissolution reaction (far from equilibrium). Variables, such as pH (acidic/alkaline medium), temperature, initial particle diameter, and Cr(VI) content in the structure were studied. For this it was necessary to: (i) synthesize and characterize a potassium jarosite sample, as well as its chromate analog and its solid solutions; (ii) select the kinetic model that describes the dissolution process and controlling stage; (iii) assess the effects of the variables on the reaction rate (pH, T, d_0_ and CrO_4_/SO_4_); (iv) determine kinetic parameters.

## Methods

### Reagents and solvents

Iron (III) sulfate *n*-hydrate, anhydrous potassium sulfate, potassium chromate, iron(III) nitrate 9-hydrate, sulfuric acid (97.9 %), hydrochloric acid (37.3 %) and sodium hydroxide were used in reagent grade. Ultrapure water (18 MΩ cm) was used in the preparation of the synthesis solutions and in all the dissolution experiments. K, Fe and Cr standards (1000 mg L^−1^) were used in the quantitative analyses and in the follow-up of the reactions. The chemical reagents were purchased from Baker, and standards from PerkinElmer Pure.

### Synthesis of solid solutions: KFe_3_[(SO_4_)_2 − X_(CrO_4_)_X_](OH)_6_

The synthesis technique for jarosite-type compounds has been widely described by different authors. We used the same technique as Reyes et al. [30] and Patiño et al. [33]. The solid solutions of KFe_3_[(SO_4_)_2 − X_(CrO_4_)_X_](OH)_6_ were synthesized by controlling a mixture of Fe_2_(SO_4_)_3_∙nH_2_O/K_2_SO_4_/K_2_CrO_4_/Fe(NO_3_)_3_∙9H_2_O in 1 l total volume. Initial Fe^3+^ content, alkali and pH are relevant factors in the synthesis of jarosite-type compounds. When the concentrations of Fe^3+^ and K^+^ in the initial solution increase, the reaction yield increases directly, so reagent concentrations well over stoichiometry were used. pH in each synthesis was adjusted between 1.2 and 1.6 with H_2_SO_4_ (20 % v/v) to avoid low yields and formation of unwanted phases [46]. A total of 7 syntheses were conducted. The solutions’ compositions are summarized in Table 1.

**Table 1:** Synthesis conditions, chemical analysis and composition of the obtained precipitates

Synthesis	Initial conditions/mol L^−1^				Elemental analysis/W %					Approximate formula
	Fe_2_(SO_4_)_3_∙nH_2_O	K_2_SO_4_	K_2_CrO_4_	Fe(NO_3_)_3_∙9H_2_O	K	Fe	Cr/CrO_4_	SO_4_	H+O^a^	
S_1_	–	–	0.2	0.2	6.90	31.44	19.12/42.67	–	18.99	[K_0_._95_(H_3_O)_0.05_]Fe_3.04_(CrO_4_)_1.99_(OH)_6.01_
S_2_	0.01	–	0.2	0.2	4.80	27.35	14.24/31.80	4.33	31.72	[K_0.61_(H_3_O)_0.39_]Fe_2.64_[(SO_4_)_0.24_(CrO_4_)_1.76_][(OH)_4.92_(H_2_O)_4.31_]
S_3_	0.05	–	0.2	0.2	5.17	28.44	9.30/20.75	17.38	28.26	[K_0.66_(H_3_O)_0.34_]Fe_2.54_[(SO_4_)_0.91_(CrO_4_)_1.09_][(OH)_4.62_(H_2_O)_3.13_]
S_4_	0.025	0.05	0.2	0.2	6.70	29.88	7.72/17.23	27.71	18.50	[K_0_._86_(H_3_O)_0.14_]Fe_2.67_[(SO_4_)_1.23_(CrO_4_)_0.77_][(OH)_5.01_(H_2_O)_0.41_]
S_5_	0.2	0.05	0.2	–	4.43	26.09	1.18/2.63	40.31	26.93	[K_0.56_(H_3_O)0._44_]Fe_2.33_[(SO_4_)_1.88_(CrO_4_)_0.12_][(OH)_3.99_(H_2_O)_3.27_]
S_6_	0.3	0.2	0.2	–	4.56	25.89	0.87/1.94	40.74	26.87	[K_0.59_(H_3_O)_0.41_]Fe_2.32_[(SO_4_)_1.91_(CrO_4_)_0.09_][(OH)_3.96_(H_2_O)_3.29_]
S_7_	0.3	0.3	–	–	5.21	27.20	–	40.51	27.08	[K_0.67_(H_3_O)_0.33_]Fe_2.43_(SO_4_)_2.11_[(OH)_4.10_(H_2_O)_3.53_]

^a^H_3_O^+^ + OH^−^ + H_2_O

### Characterization of solid solutions: KFe_3_[(SO_4_)_2 − X_(CrO_4_)_X_](OH)_6_

For the elemental analysis, it was necessary to dissolve a sample of each of the obtained precipitates (1 g) in a 1:1 solution of water-concentrated hydrochloric acid. The solutions were analyzed in a PerkinElmer Analyst 200 atomic absorption spectrometer (AAS) to determine K, Fe and Cr. SO_4_^2−^ was determined by gravimetric analysis as BaSO_4_. The obtained solids were also analyzed by X-ray diffraction (XRD) with a SIEMENS D-500 using Cu Kα radiation (1.54056 Å). Morphology of the solids was examined using a JEOL JSM-5900LV scanning electron microscope (SEM) equipped with a noran energy dispersive X-ray spectrometer (EDS). The precipitates were also characterized using a Perkin Elmer–Frontier fourier transform infrared (FT–IR) spectrometer equipped with an attenuated total reflectance (ATR) accessory to confirm water in the crystal structure and to validate the presented formulae. The obtained precipitates were wet-sieved to separate them by particle size with the Tyler mesh size series (USA Standard Testing Sieve, ASTME-11 specifications). The used mesh sizes were the following: 120 (d_0_ ≥ 125 μm), 170 (125 < d_0_ ≥ 90 μm), 200 (90 < d_0_ ≥ 75 μm), 270 (75 < d_0_ ≥ 53 μm), 325 (53 < d_0_ ≥ 44 μm), 400 (44 < d_0_ ≥ 38), and 500 (38 < d_0_ ≥ 25 μm).

### Dissolution experiments in acidic/alkaline (H_3_O^+^/OH^−^) medium

0.2 ± 0.0001 g of the synthesized solid were used in an initial volume of 500 ± 0.0002 mL for all the experiments. The acidic conditions were obtained through dilutions of concentrated HCl (37.3 % purity), and the alkaline conditions through direct NaOH pellet weighting. For low concentrations, it was necessary to conduct dilutions of an initial NaOH 0.1 mol L^−1^ solution. The solution of each experiment was placed in a (Pyrex) glass reactor and set on a heating plate with automatic temperature control and mechanical stirring at a rate of 750 min^−1^ to avoid particle fragmentation. In all the decomposition experiments pH was kept constant by adding low volumes of a concentrated NaOH or HCl solution correspondingly (1.0 mol L^−1^). Progress of the dissolution reaction was monitored by taking samples of the solution (5.0 ± 0.01 mL) at different times established according to the total reaction time (t_r_) of each experiment (≈20 samples per experiment). Each sample was analyzed for potassium by AAS (it was previously filtered with Whatman # 42 filter paper to remove solid residues). Alterations due to sampling and reagent addition were corrected through mass balance. The effects of H_3_O^+^/OH^−^ concentration, temperature (T), particle initial diameter (d_0_) and Cr(VI) proportion in the structure (SO_4_^2−^/CrO_4_^2−^), were studied by changing a parameter and keeping the other three constant in each experiment. pH readings are essential in this work, so it was intermittently measured for each experiment in the bulk of the solution using an Orion 3 star pH-meter equipped with a thermo ultra sure flow electrode with a reading precision of pH ± 0.01, and use range of 0–14 at a maximum temperature of 100 °C. It also has an automatic temperature compensation electrode with an accuracy of T ± 0.5 °C. During the average pH measurement time (≈30 s) the loss of filling solution is minimal and can be considered that the filling solution does not contaminate the sample.

## Results

### Synthesis and characterization

The syntheses under the conditions in Table 1 produce, from 30 g for the chromate analog of potassium jarosite (S_1_) (this output grows as the substitution of SO_4_ in the structure increases), to 70 g for potassium jarosite (S_7_), where the substitution of *Z*-site by S(VI) is total. A possible explanation for the preferential incorporation of sulfate is that in synthetic acidic solutions, Cr(VI) is mainly present as HCrO_4_^−^, and the CrO_4_^2−^ concentration, which is incorporated into the precipitated solids, is low compared to the total concentration of Cr(VI) in the solution. On the other hand, sulfur is preferentially present as SO_4_^2−^ [4]. Precipitate color varies from red in KFe_3_(CrO_4_)(OH)_6_ to the characteristic yellow color of jarosite in S_7_. The chemical analysis shows that, of the seven syntheses we carried out, only S_1_ has a stoichiometry close to the ideal formula of jarosite-type compounds MFe_3_(ZO_4_)_2_(OH)_6_, with a molar proportion K/Fe/CrO_4_/OH of 0.95/3.04/1.99/6.01, compared to the ideal 1/3/2/6 proportion. Deviations from the ideal formula in syntheses S_2_ to S_7_ are due to K^+^ deficiencies attributed to the substitution of H_3_O^+^ and the deficiency of Fe^3+^, which is compensated by the conversion of OH^−^ to H_2_O. These deficiencies have also been observed on other natural and synthetic jarosite-type compounds [15, 19, 20, 27]. By considering the molar relations K^+^ + H_3_O^+^ = 1, SO_4_^2−^ + CrO_4_^2−^ = 2 and OH^−^ = 3Fe^3+^ −3, it is possible to calculate the approximate formulas shown on Table 1.

**Fig. 1:**
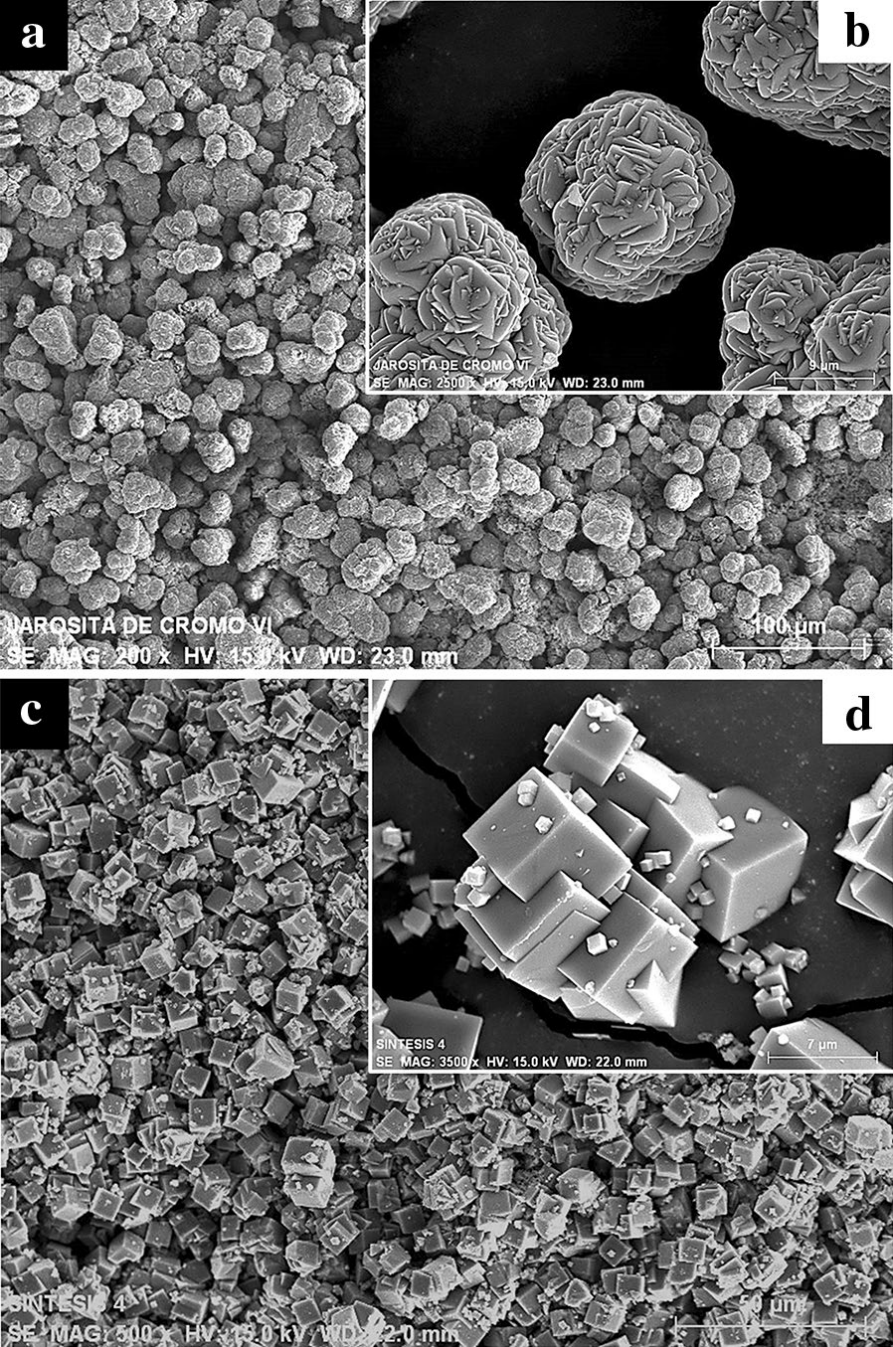
SEM images of the synthesized solids. **a** Particles synthesized in S_2_; similar characteristics are observed from S_2_ to S_7_. **b** *Spheroidal* aggregate obtained from S_2_ to S_7_. **c** Particles synthesized in S_1_. **d** *Cubic* euhedral aggregate obtained in S_1_. From synthesis S_2_ to S_7_, the precipitates are mainly composed of *spheroidal* aggregates, which are typical of synthetic jarosite-type compounds

SEM images show that, for syntheses S_2_ to S_7_, the precipitates are mainly composed of spheroidal aggregates with a diameter between 20 and 90 µm (Fig. 1a), which are typical of synthetic jarosite-type compounds. Regarding the chromate analog of potassium jarosite, S_1_ is composed of intergrown crystals with cubic euhedral morphology, as seen on Fig. 1c, with crystallite sizes ranging from 1 to 10 µm for all the synthesized solids (Fig. 1b–d). EDS analyses of all the precipitates show uniform concentrations of K/Fe/S/Cr.

**Fig. 2:**
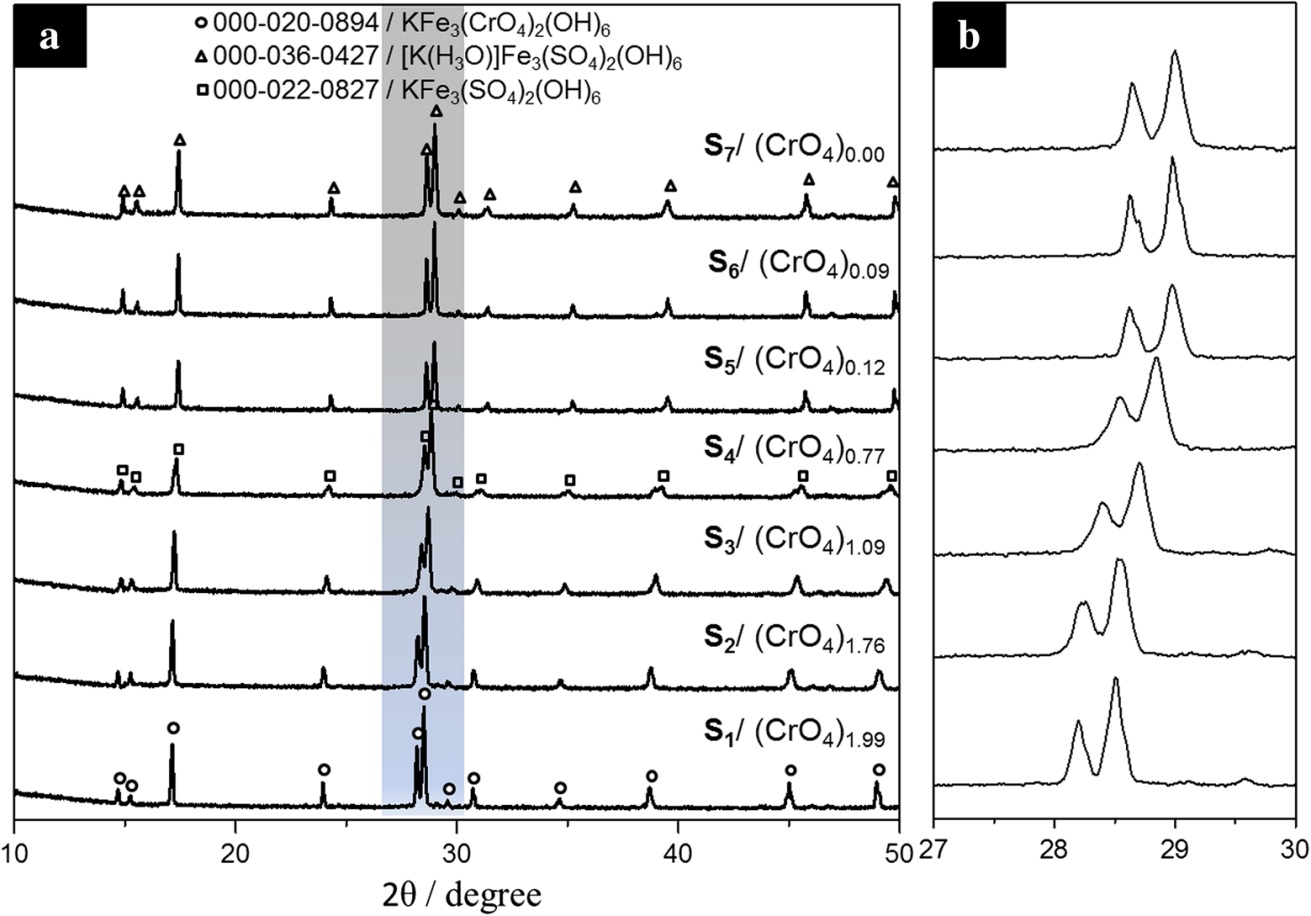
X-ray spectra obtained for the synthesized solids. **a** All the signals were identified as jarosite-type compounds **b** Close up view of the main X-ray diffraction peaks of the solid solution KFe_3_[(SO_4_)_2 − X_(CrO_4_)_X_](OH)_6_ (*shaded area* on Fig. 2a)

The XRD patterns obtained from the precipitates were compared to those of the International Center for Diffraction Data-Powder Diffraction Files. The results are presented in Fig. 2a. All of the synthesized solids were identified as jarosite-type compounds. S_1_ was compared to the pattern KFe_3_(CrO_4_)(OH)_6_ reported on card ICDD-PDF 000-020-0894, and all the peaks were identified as KFe_3_(CrO_4_)(OH)_6_. Precipitates S_2_, S_3_, S_5_, S_6_ and S_7_ were similar with card ICDD-PDF 000-036-0427, which corresponds to [K(H_3_O)]Fe_3_(SO_4_)(OH)_6_. As observed in Table 1, in the range between S_2_ and S_6_, S_4_ is the precipitate with the least amount of H_3_O^+^ in its structure, so all the XRD signals of this solid were identified as KFe_3_(SO_4_)(OH)_6_ (ICDD-PDF 000-022-0827). The absence of unidentified peaks indicates that there are no other crystal phases at detectable levels in any of the synthesized solids. A slight offset in the main signals of the solids can be observed as Cr content grows (Fig. 2b) towards smaller angles (2θ), representing slightly larger d-spacings of the jarosite’s chromate analog, because the unit cell volume of KFe_3_(CrO_4_)(OH)_6_ is slightly higher than the volume of KFe_3_(SO_4_)(OH)_6_. This slight change indicates a continuous solid solution instead of a two-phase mixture, because in case of a mixture, different groups of peaks of each phase would be present, and the intensity of those peaks would be the separate function of each phase’s fraction in the mixture [4].

**Fig. 3:**
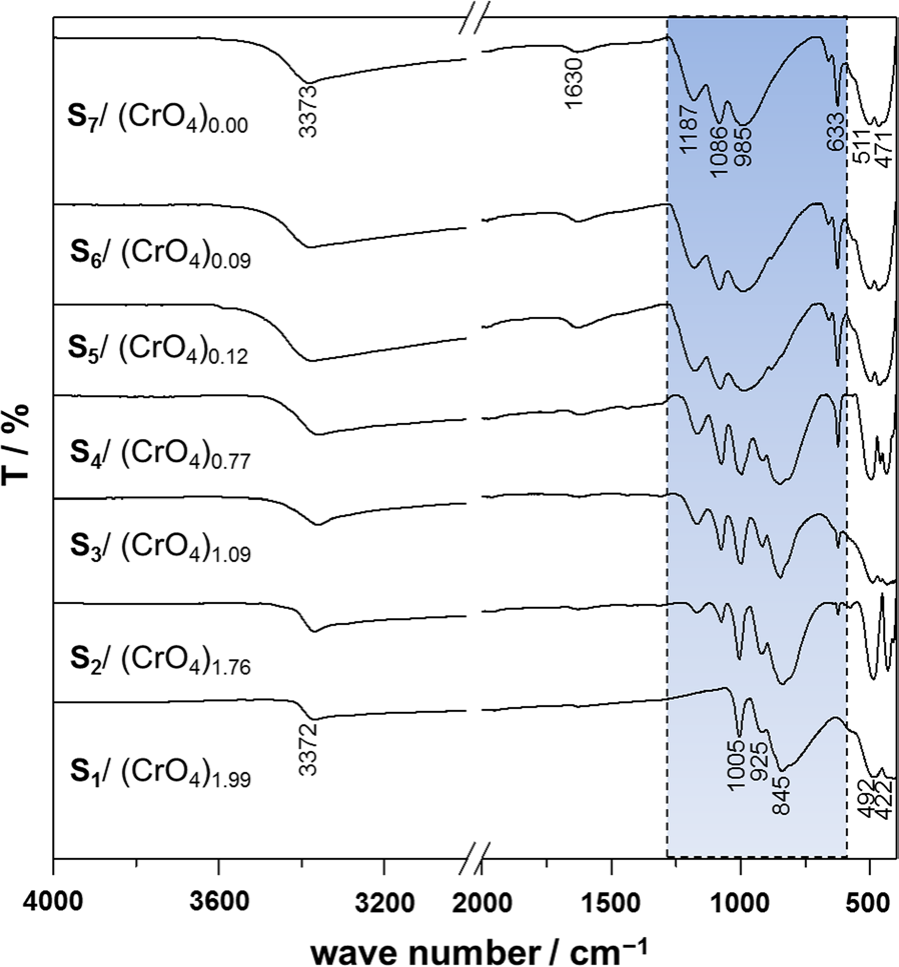
FT-IR spectrum in transmittance of the solid solutions KFe_3_[(SO_4_)_2 − X_(CrO_4_)_X_](OH)_6_. The main vibration bands in the spectra have been *highlighted*. Results confirm the stoichiometry of the compounds shown in Table 1

The FT-IR results (Fig. 3) for the solids synthesized in this study are very similar to other previously reported studies on natural and synthetic jarosite-type compounds [2, 11, 27, 47]. The two most intense peaks, near 1086 and 1187 cm^−1^, occurred due to the stretching vibration ν_3_, and the double peak observed near 633 cm^−1^ occurred due to the bending vibration mode ν_4_, both in SO_4_^2−^. The band that appears at approximately 3372 cm^−1^ is mainly due to the stretching mode of OH^−^, and it also includes vibration modes of water, especially for synthetic samples where there is a substitution by H_3_O^+^ in the cationic position. The water band appears at approximately 1630 cm^−1^, which is similar in all of the synthesized solids. Besides, in S_1_, where the substitution of CrO_4_^2−^ is total, it is possible to observe the mode ν_3_ of CrO_4_^2−^ (925 and 845 cm^−1^) and the deformation of OH^−^ at 1005 cm^−1^. Between S_1_ (492 and 422 cm^−1^) and S_7_ (511 and 471 cm^−1^) the vibration modes of the octahedral coordination of FeO_6_ can be observed. In the shaded area on Fig. 3 it is possible to observe how the substitution of CrO_4_^2−^ by SO_4_^2−^ takes place, and the distinctive vibration modes of both species in one same compound are clearly visible between S_2_ and S_6_, which confirms the solid solution between KFe_3_(CrO_4_)(OH)_6_ and KFe_3_(SO_4_)(OH)_6_. XRD and FT-IR results confirm the stoichiometry of the synthesized solids (shown in Table 1).

### Kinetic model selection and stage controlling the dissolution rate

**Fig. 4:**
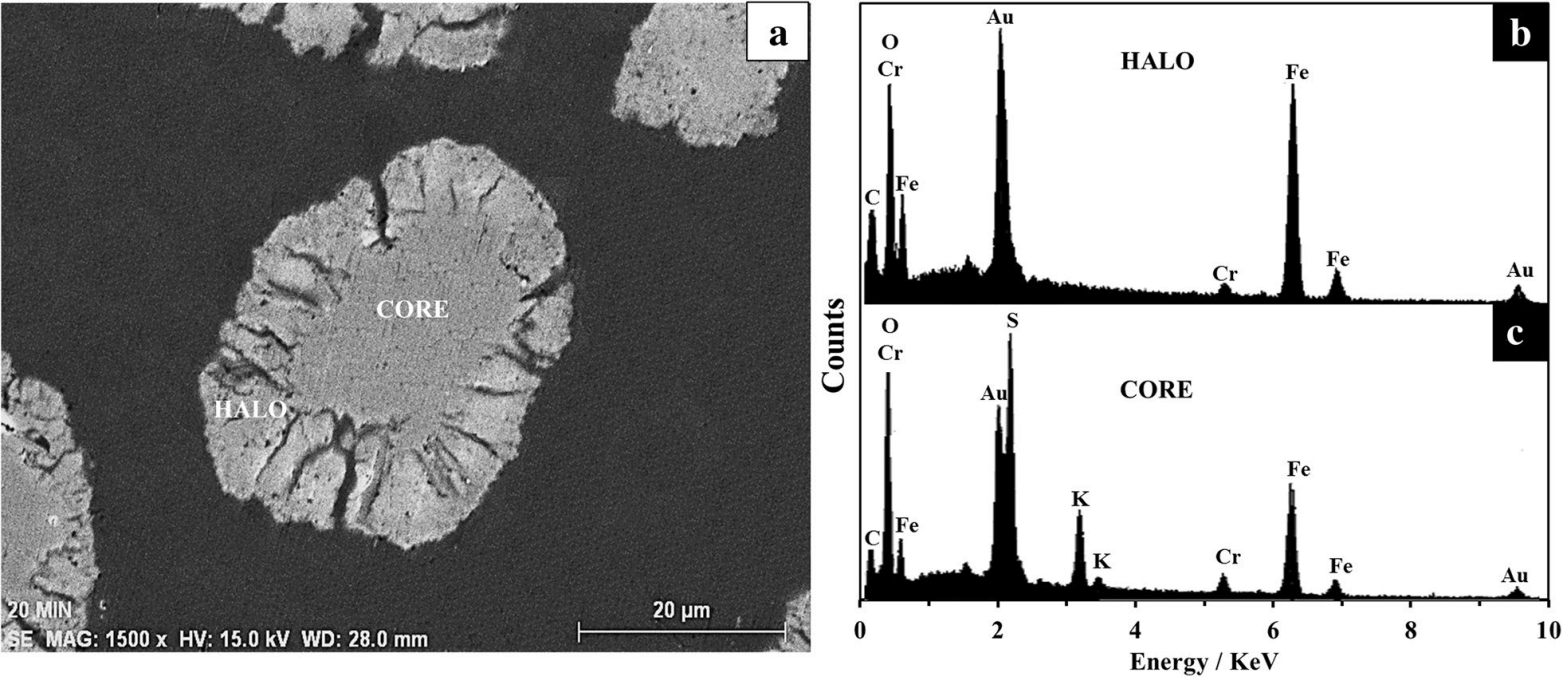
SEM–EDS results of a partially decomposed particle in OH^−^ medium. **a** SEM image of a partially decomposed particle in OH^−^medium (0.05 mol L^−1^, T = 30 °C, pH = 12.44, d_0_ = 38–44 µm, CrO_4_^−2^ = 0.77 mol, t_r_ = 20 min). **b** EDS analysis corresponding to the halo in Fig. 4a. **c** EDS analysis corresponding to the core in Fig. 4a. Results show that the unreacted core kinetic model with formation of ashes describes the dissolution process of KFe_3_[(SO_4_)_2 − X_(CrO_4_)_X_](OH)_6_ in OH^−^ medium

**Fig. 5:**
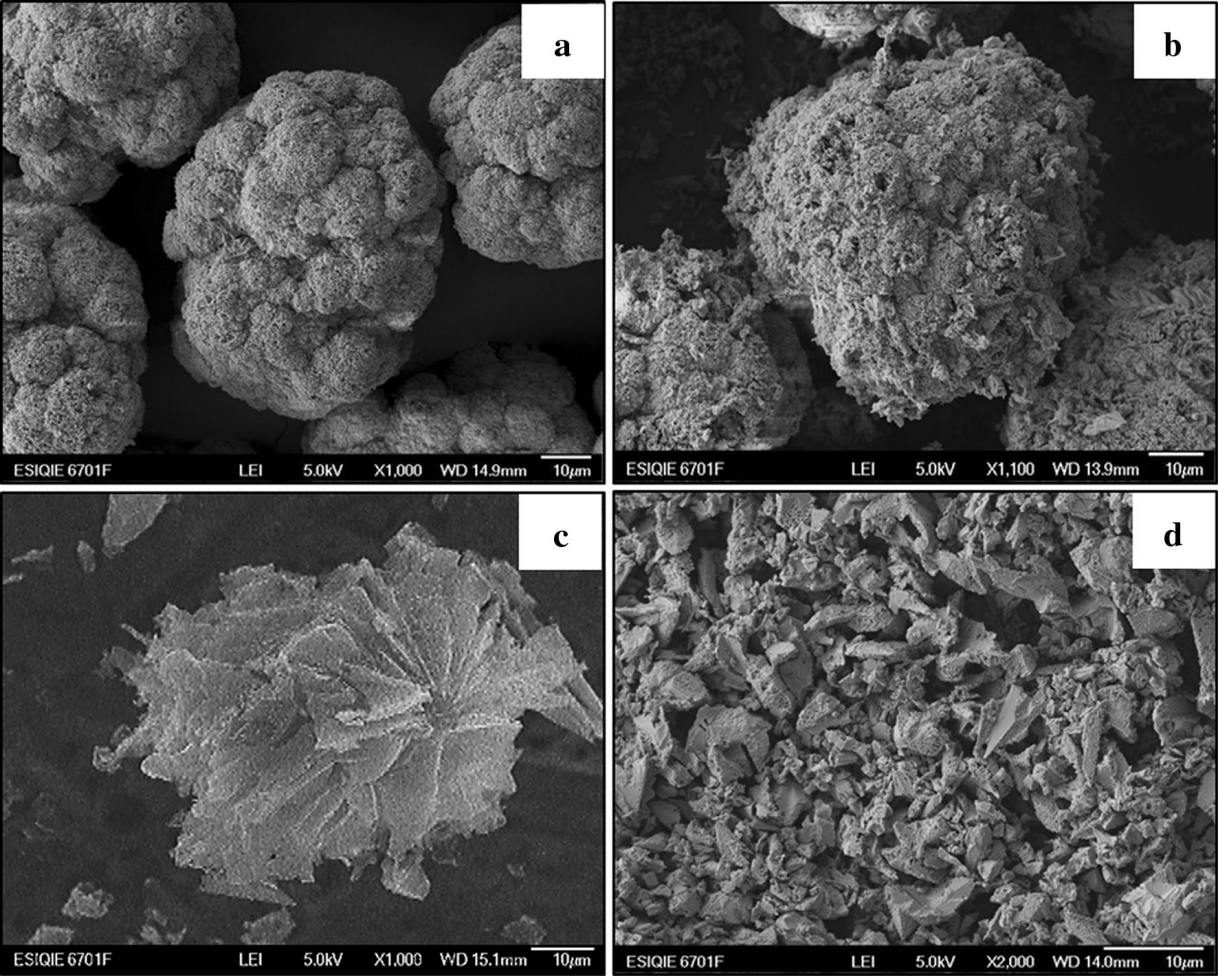
SEM image of partially decomposed particles in H_3_O^+^ medium (0.01 mol L^−1^, T = 50 °C, pH 2.01, d_0_ = 38–44 µm, CrO_4_^−2^ = 0.77 mol); **a** t_r_ = 500 min; **b** t_r_ = 1000 min; **c** t_r_ = 1500 min; **d** t_r_ = 2000 min. Results show that the unreacted core kinetic model without formation of an *ash layer* describes the dissolution process of KFe_3_[(SO_4_)_2 − X_(CrO_4_)_X_](OH)_6_ in H_3_O^+^ medium

Several samples of S_4_ were treated with an HCl and NaOH solution at different T and d_0_ conditions. The solutions were analyzed for K by AAS at different time intervals, and the remaining solids were characterized by SEM–EDS and XRD. Levenspiel [48] states that, for non-catalytic reactions of solid particles with a surrounding fluid, two kinetic models are considered: the progressive conversion model, where the reacting fluid penetrates and reacts throughout the particle, and the unreacted core model, where the reaction first takes place on the external surface of the solid particle, and then it moves inside the solid, leaving completely transformed material (ashes) behind. Therefore, during the reaction there will be an untransformed material core, whose size will decrease as the reaction progresses. For the dissolutions in OH^−^ medium, when cutting and examining the transversal section of particles that have partially reacted (Fig. 4a), it is possible to observe solid material that has not reacted (core), surrounded by a halo (ash layer). EDS analyses show the presence of Fe, Cr and O in the halo (Fig. 4b), while K^+^ and SO_4_^2−^ have diffused into the solution, while it is possible to notice the presence of K, Fe, S, Cr and O in the core (Fig. 4c), indicating that it has not reacted. The Au and C signals appear because the sample was fixed in resin and covered in gold. Regarding the dissolutions in H_3_O^+^ medium, there is no formation of ash layer, because the reaction products are soluble and also there are detachments of flakes. Therefore the particle’s size decreases during the reaction until it totally disappears. This process is represented in Fig. 5a–d. EDS analyses of the solids for each stage show uniform concentrations of K/Fe/S/Cr/O. As it can be noticed, the unreacted core model for spherical particles with the formation of an ash layer satisfactorily describes the dissolution process of jarosite-type compounds in OH^−^ media and most of the reactions in acidic media, specifically the developed at high concentrations of H_3_O^+^. In the case of the reactions developed at low concentrations of H_3_O^+^, the model that describes the process is the unreacted core model for spherical particles without the formation of an ash layer.

The process of determining the kinetics and rate controlling stages in a solid–fluid reaction is done by following the conversion of solid particles and by observing how their size, temperature, medium concentration and reaction time affect the conversion. Conversion *X* is a dimensionless number that it is the amount of substance that has reacted, and for the purpose of this work, it is possible to calculate *X* as follows:

1 $$ X = \frac{{A_{t} }}{{A_{\tau } }} $$

where *X* is the KFe_3_[(SO_4_)_2 − X_(CrO_4_)_X_](OH)_6_ fraction that has reacted, *A*_*t*_ is the amount of K in the solution at a given time *t*, and *A*_*τ*_ is the amount of K when the reaction has reached steady state.

**Fig. 6:**
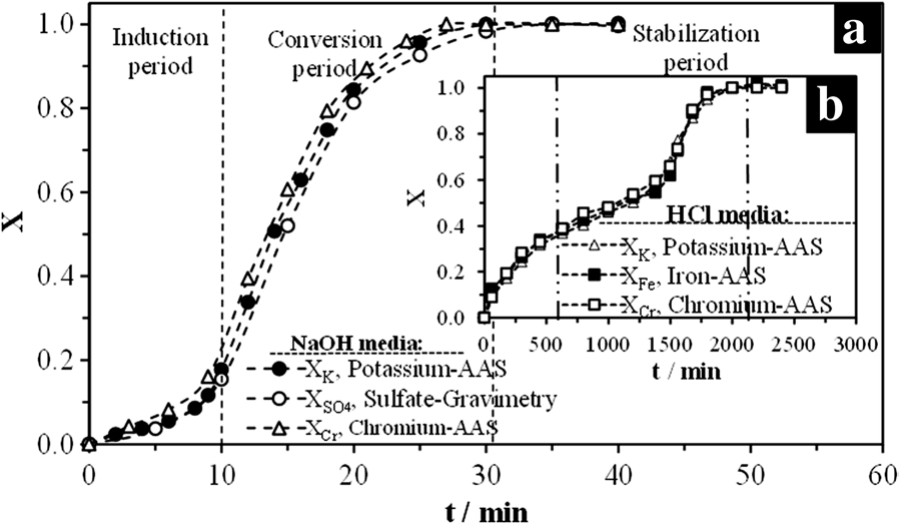
S_4_ dissolution curves; **a** OH^−^ medium (0.05 mol L^−1^, T = 30 °C, pH 12.44, d_0_ = 38–44 µm, CrO_4_^−2^ = 0.77 mol); **b** H_3_O^+^ medium (0.01 mol L^−1^, T = 50 °C, pH 2.01, d_0_ = 38–44 µm, CrO_4_^−2^ = 0.77 mol)

**Fig. 7:**
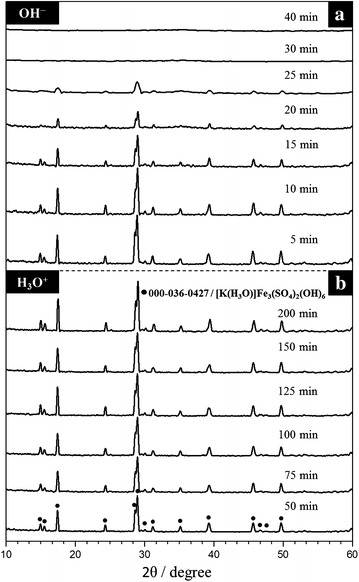
X ray diffraction patterns at different reaction times; **a** OH^−^ medium (0.05 mol L^−1^, T = 30 °C, pH 12.44, d_0_ = 38–44 µm, CrO_4_^−2^ = 0.77 mol); **b** H_3_O^+^ medium (0.3 mol L^−1^, T = 50 °C, pH = 0.50, d_0_ = 38–44 µm, CrO_4_^−2^ = 0.77 mol)

Figure 6a shows the effect of time on the conversion of K^+^ and SO_4_^2−^into a product for the dissolution of S_4_ in OH^−^ medium. It is possible to notice an induction period in the dissolution curve, where there is no change in color or in the morphology of the particles. This stage is related to the difficulty of absorption of OH^−^ ions on the particles’ surface to form active sites; the duration of this period is known as induction time (t_ind_). The end of the induction period is identified by a change in color, going from red to grey, and it indicates the establishment of a reaction front, where the concentration of K^+^ and SO_4_^2−^and CrO_4_^2−^ progressively increase (progressive conversion) until reaching a steady state. This indicates the end of the dissolution reaction (stabilization period). The shape of the curve presented in Fig. 6a is common for most of the dissolution reactions conducted for this study in both reaction media. For the reactions in acidic medium conducted at low [H_3_O^+^] (<0.07 mol L^−1^), a dissolution curve similar to that presented in Fig. 6b was obtained. It can be observed that, after 1250 min of reaction, there is a sharp change in the slope, which indicates a change in the reaction rate. This can be attributed to the fragmentation of particles, as observed in Fig. 5d, where particle diameter shrinks drastically, thus increasing the contact area, which in turn increases the reaction rate. This change appears to be very evident in these reactions because they are slow compared to those conducted at high temperatures and pH. The reaction progress followed by XRD in OH^−^medium, Fig. 7a, corresponds to the data presented in Fig. 6a. It can be noticed that, while the concentrations of K^+^ and SO_4_^2−^increase in the solution, the reflection intensities of the XRD peaks gradually decrease until they disappear; the solid residues are amorphous and do not evolve into new crystal phases. Decomposition in acidic medium (Fig. 7b) at low concentration of [H_3_O^+^] (≤0.07 mol L^−1^ of HCl ≈ pH = 1.13 for T = 30 and 50 °C) results in an incomplete solid dissolution using steady state conditions for calculation of the conversion. The residual solid was identified by DRX and SEM–EDS as KFe_3_[(SO_4_)_2 − X_(CrO_4_)_X_](OH)_6_; with no evidence of the formation of secondary phases. Also in Fig. 6 was demonstrated that can be use any value of conversion of the present ions for the calculation of constant rates for both reaction media (Fe, Cr, SO_4_), since it was demonstrated to have the same dissolution rate. For convenience only are presented the values of conversion of potassium.

The controlling stage in a solid–fluid reaction is that which presents higher resistance. In the unreacted shrinking core model for spherical particles with formation of solid products, two stages can be slow: the chemical reaction in the interface between the unreacted core and the ash halo, and the diffusion of reagents and products through the ash halo [48]. When the chemical reaction is slow, compared to the rate of matter transportation, the kinetic equation that describes the process is the following:

2 $$ 1 - \left( {1 - X} \right)^{1/3} = k_{exp} t $$

On the other hand, when the diffusion through the ash halo is slow, the equation that describes the process is the following:

3 $$ 1 - 3\left( {1 - X} \right)^{2/3} + 2\left( {1 - X} \right) = k_{exp} t $$

In addition, in the unreacted shrinking core model for spherical particles without formation of an ash halo, when the matter transportation is the controlling stage, the equation that describes the process is the following:

4 $$1 - {\left( {1 - X} \right)^{{2 \mathord{\left/
 {\vphantom {2 3}} \right.
 \kern-\nulldelimiterspace} 3}}} = {k_{exp}}t $$

**Fig. 8:**
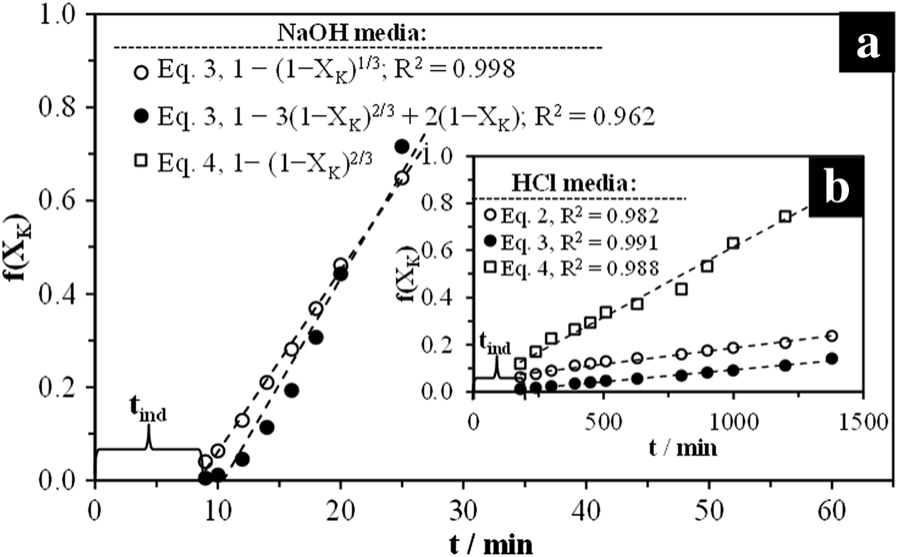
Assessment of Eqs. 2, 3 and 4 corresponding to the different controlling stages in the unreacted core model. **a** OH^−^ medium; **b** H_3_O^+^ medium. X_K_ is the potassium fraction in the solution. In the graphics, the slope is k_exp_ and the intersection with t is the induction time (t_ind_). k_exp_ and the inverse of t_ind_ are the rate constants for every stage (both in min^−1^). The time necessary to achieve any degree of conversion is equal to the sum of the times required for each kinetic stage

In Eqs. 2, 3 and 4, *X* is the KFe_3_[(SO_4_)_2 − X_(CrO_4_)_X_](OH)_6_ decomposed fraction, *k*_*exp*_ is the experimental rate constant and *t* is time [49]. For the confirmation of any of the three equations, an experiment was conducted where [H_3_O^+^]/[OH^−^], T, d_0_ and Cr content in the structure were kept constant, and the conversion was determined at different times. A representation of Eqs. 2, 3 and 4 in function of time should be linear, the slope is *k*_*exp*_ and the intersection with *t* is the induction time (*t*_*ind*_), which represents the induction period duration. [30, 32, 49]. Figure 8 corresponds to the assessment of Eqs. 2 and 3 with the data obtained in Fig. 6. As it can be noticed, the assessment of Eq. 2, which corresponds to a chemical control, matches the linear requirement for the decomposition in OH^−^ medium very well. For the decomposition in H_3_O^+^ medium at low [H_3_O^+^], the equation that best matches the linear requirement is, surprisingly, Eq. 3, which corresponds to the unreacted core model with diffusion control in the solid product halo, and according to SEDM–EDS results, there is no formation of ashes under these reaction conditions. Therefore, the hypotheses on which the model is based may not completely describe the real process, e.g. the reaction can occur along a diffused front instead of doing it on a defined surface between the unreacted solid, the ashes or the fluid layer, so it corresponds to an intermediate behavior between the two mentioned models and controlling stages [48]. Consequently, the unreacted core model with its respective controlling stages is the model accepted to describe the dissolution process of potassium jarosite, its chromate analog and its solid solutions in [H_3_O^+^]/[OH^−^] media after the induction period.

Therefore, the experimental rate constant of the reaction for a chemical control can be defined as follows:

5 $$ k_{exp} = \frac{{bk_{q} C_{A}^{n} }}{{\rho_{J} d_{0} }} $$

where *b* is a stoichiometric coefficient, *k*_*q*_ is the reaction rate chemical constant, *C*_*A*_ is the reactant concentration ([H_3_O^+^]/[OH^−^]), *n* is the reaction order, *ρ*_*J*_ is the molar density of S_4_ and *d*_*0*_ is the particle initial diameter in µm. In the case of the experimental rate constant of the reaction for a control by matter transportation with formation of ash halo, it is defined as follows:

6 $$ k_{exp} = \frac{{2bD_{e} C_{A} }}{{\rho_{J} d_{0}^{2} }} $$

where *b* is a stoichiometric coefficient, *D*_*e*_ is the effective diffusion coefficient in a porous structure (cm s^−2^), *C*_*A*_ is the reactant concentration ([H_3_O^+^]), *ρ*_*J*_ is the molar density of S_4_, and *d*_*0*_ is the particle initial diameter in µm. Notice that the reaction order in this equation is *n* = 1.

### Experimental data and kinetic parameters

**Fig. 9:**
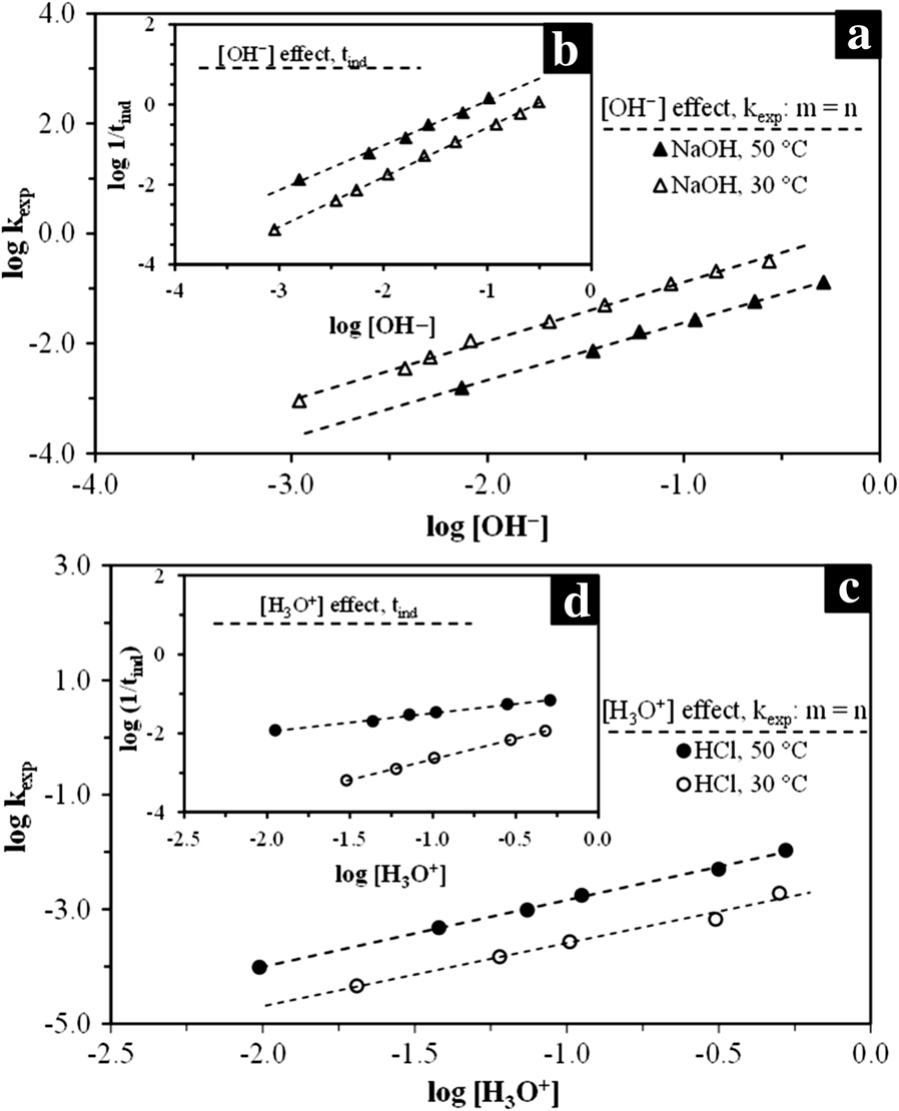
Plots for the determination of the reaction order at 30 and 50 °C. **a** Progressive conversion period in OH^−^ medium; **b** induction period in OH^−^ medium; **c** Progressive conversion period in H_3_O^+^ medium; **d** induction period, H_3_O^+^ medium

**Fig. 10:**
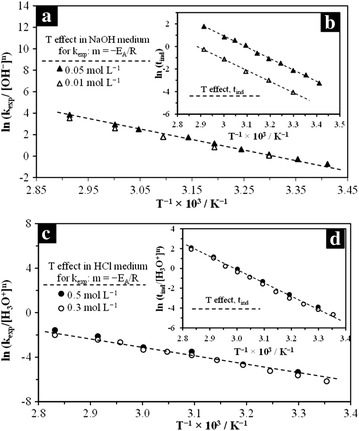
Arrhenius plots for the determination of the activation energy. **a** Progressive conversion period, OH^−^ medium; **b** induction period in OH^−^ medium; **c** progressive conversion period, H_3_O^+^ medium; **d** induction period, H_3_O^+^ medium. The concentrations used for OH^−^ medium were 0.05 and 0.01 mol L^−1^ NaOH; 0.5 and 0.3 mol L^−1^ HCl were used for H_3_O^+^medium

Figure 6a and b shows the dissolution curve shape of all the experiments conducted in both reaction media. All the experimental data are summarized in Table 2 for OH^−^ medium, and in Table 3 for the reactions in H_3_O^+^ medium. [OH^−^] in Table 3 was determined through the initial pH and the ionization constant of water (k_w_) at working temperature in each experiment [50]. After calculating the conversion *X* for all the experimental data and applying Eq. 2, and in its case, Eq. 3 to obtain *k*_*exp*_ and *t*_*ind*_, it is possible to obtain the reaction order *n* and activation energy *E*_*A*_ (kinetic parameters), as reported by Patiño et al. [33, 34], 2013 for each medium and controlling stage for dissolutions of jarosite type-compounds. Calculations are shown in Figs. 9a, b and 10a, b for reaction order and for activation energy, respectively. The results of the kinetic parameters calculated are summarized in Table 4.

**Table 2:** Conditions and calculation results of the dissolution experiments of KFe_3_[(SO_4_)_2 − X_(CrO_4_)_X_](OH)_6_ in medium H_3_O^+^, tr is the total reaction time of each experiment (until steady state is obtained)

[HCl]/mol L^−1^	pH	T/K	CrO_4_/mol^a^	[H_3_O^+^]/mol L^−1^	d_0_/µm^b^	t_ind_/min	k_exp_/min ^−1^	t_r_/min
0.50	0.28	323.15	0.77	0.5248	38	5.50	0.01070	50
0.30	0.50	323.15	0.77	0.3162	38	18.44	0.00830	130
0.10	0.95	323.15	0.77	0.1122	38	29.88	0.00177	420
*0.07*	*1.13*	*323.15*	*0.77*	*0.0741*	*38*	*34.06*	*0.00097*	*520*
*0.04*	*1.42*	*323.15*	*0.77*	*0.0380*	*38*	*53.50*	*0.00048*	*750*
*0.01*	*2.01*	*323.15*	*0.77*	*0.0098*	*38*	*84.47*	*0.00010*	*1800*
0.50	0.30	303.15	0.77	0.5012	38	75.70	0.00191	390
0.30	0.51	303.15	0.77	0.3090	38	148.58	0.00067	1320
0.10	0.99	303.15	0.77	0.1023	38	458.77	0.00027	2340
*0.06*	*1.22*	*303.15*	*0.77*	*0.0603*	*38*	*1033.32*	*0.00015*	*10320*
*0.02*	*1.69*	*303.15*	*0.77*	*0.0204*	*38*	*2740.02*	*0.00005*	*13500*
0.30	0.55	298.15	0.77	0.2818	38	191.25	0.0004	2280
0.30	0.55	308.15	0.77	0.2818	38	66.27	0.0011	1000
0.30	0.54	313.15	0.77	0.2884	38	36.58	0.0019	750
0.30	0.54	318.15	0.77	0.2884	38	19.00	0.0030	285
0.30	0.53	323.15	0.77	0.2951	38	8.69	0.0048	120
0.30	0.52	333.15	0.77	0.3020	38	2.52	0.0083	47
0.30	0.51	338.15	0.77	0.3090	38	1.41	0.0162	40
0.30	0.50	343.15	0.77	0.3162	38	0.61	0.0210	35
0.30	0.49	353.15	0.77	0.3236	38	0.25	0.0326	30
0.50	0.33	303.15	0.77	0.468	38	109.80	0.00193	390
0.50	0.31	313.15	0.77	0.490	38	29.56	0.00407	150
0.50	0.27	323.15	0.77	0.537	38	7.24	0.0143	50
0.50	0.25	333.15	0.77	0.562	38	1.98	0.0223	30
0.50	0.22	343.15	0.77	0.603	38	0.54	0.0651	12
0.50	0.21	353.15	0.77	0.617	38	0.21	0.1173	8
0.30	0.52	303.15	0.77	0.3020	25	18.13	0.0083	130
0.30	0.52	303.15	0.77	0.3020	38	18.44	0.0056	130
0.30	0.53	303.15	0.77	0.2951	44	20.83	0.0048	130
0.30	0.53	303.15	0.77	0.2951	53	17.64	0.0039	130
0.30	0.53	303.15	0.77	0.2951	75	15.21	0.0028	130
0.30	0.52	303.15	1.99	0.3020	38	5.61	0.0060	165
0.30	0.52	303.15	1.57	0.3020	38	5.23	0.0059	165
0.30	0.53	303.15	1.02	0.2951	38	5.70	0.0059	160
0.30	0.52	303.15	0.77	0.3020	38	4.94	0.0056	160
0.30	0.53	303.15	0.12	0.2951	38	6.04	0.0058	165
0.30	0.53	303.15	0.09	0.2951	38	8.56	0.0059	165
0.30	0.52	303.15	0.00	0.3020	38	6.58	0.0059	160

Italic data indicates conditions and results with change in the controlling stage (or resistance), passing from a process controlled by the chemical reaction to a process controlled by the mass transport in the ash layer

^a^SO_4_^2−^ + CrO_4_^2−^ = 2

^b^Mesh diameter

**Table 3:** Conditions and calculation results of the dissolution experiments of KFe_3_[(SO_4_)_2 − X_(CrO_4_)_X_](OH)_6_ in OH^−^ medium

[NaOH]/mol L^−1^	pH	T/K	CrO_4_/mol^a^	[OH^−^]/mol L^−1^	d_0_/µm^b^	t_ind_/min	k_exp_/min ^−1^	t_r_/min
0.100	12.29	323.15	0.77	0.1023	38	0.7	0.5929	2.5
0.050	12.14	323.15	0.77	0.0724	38	1.6	0.2138	4.5
0.030	11.81	323.15	0.77	0.0339	38	3.2	0.1284	10.0
0.010	11.49	323.15	0.77	0.0162	38	6.8	0.0597	20.0
0.006	11.14	323.15	0.77	0.0072	38	16.5	0.0346	30.0
0.001	10.37	323.15	0.77	0.0012	38	75.0	0.0197	115.0
0.300	13.36	303.15	0.77	0.3342	38	0.86	0.2735	4.0
0.200	13.15	303.15	0.77	0.2061	38	1.69	0.1474	5.0
0.100	12.94	303.15	0.77	0.1271	38	3.18	0.0962	16.0
0.050	12.45	303.15	0.77	0.0397	38	8.63	0.0397	30.0
0.025	12.18	303.15	0.77	0.0221	38	19.04	0.0207	50.0
0.010	11.88	303.15	0.77	0.0111	38	55.2	0.0082	160.0
0.007	11.67	303.15	0.77	0.0065	38	137.49	0.0051	280.0
0.003	11.38	303.15	0.77	0.0035	38	253.74	0.0038	420.0
0.001	10.79	303.15	0.77	0.0009	38	1342.41	0.0011	2100.0
0.050	12.88	293.15	0.77	0.05200	38	26.0	0.0200	70.0
0.050	12.62	298.15	0.77	0.04200	38	12.5	0.0251	40.0
0.050	12.18	308.15	0.77	0.03100	38	4.4	0.0455	18.0
0.050	12.04	313.15	0.77	0.03100	38	3.5	0.0791	14.0
0.050	11.92	318.15	0.77	0.03300	38	2.0	0.1464	10.0
0.050	11.87	323.15	0.77	0.03900	38	1.7	0.1881	7.0
0.050	11.51	328.15	0.77	0.02300	38	0.8	0.2103	3.7
0.050	11.39	333.15	0.77	0.02300	38	0.4	0.3248	2.8
0.050	11.04	343.15	0.77	0.01700	38	0.1	0.5794	2.3
0.010	11.88	303.15	0.77	0.01096	38	58.0	0.0082	160.0
0.010	11.79	313.15	0.77	0.01778	38	20.1	0.0299	51.0
0.010	11.34	323.15	0.77	0.01148	38	9.1	0.0484	24.0
0.010	10.91	333.15	0.77	0.00759	38	3.1	0.0705	16.0
0.010	10.68	343.15	0.77	0.00741	38	1.3	0.1752	5.0
0.050	12.53	303.15	0.77	0.049	75	7.5	0.02	24.0
0.050	12.54	303.15	0.77	0.050	53	9.81	0.0311	27.0
0.050	12.55	303.15	0.77	0.051	44	8.63	0.034	25.0
0.050	12.53	303.15	0.77	0.049	38	9.3	0.0397	30.0
0.050	12.52	303.15	0.77	0.048	25	9.1	0.0632	28.0
0.050	12.54	303.15	1.99	0.0500	38	6.9	0.0373	27.0
0.050	12.68	303.15	1.57	0.0555	38	2.3	0.0358	25.0
0.050	12.68	303.15	1.02	0.0555	38	2.8	0.0373	25.0
0.050	12.45	303.15	0.77	0.0411	38	8.6	0.0397	30.0
0.050	12.53	303.15	0.12	0.049	38	4.2	0.0369	24.0
0.050	12.48	303.15	0.09	0.0555	38	6.9	0.0367	27.0
0.050	12.69	303.15	0.00	0.0568	38	4.3	0.0392	25.0

^a^SO_4_^2−^ + CrO_4_^2−^ = 2

^b^Mesh diameter

**Table 4:** Kinetic parameters calculated in the dissolution experiments of KFe_3_[(SO_4_)_2 − X_(CrO_4_)_X_](OH)_6_ in medium H_3_O^+^ and OH^−^ for induction and conversion periods

Kinetic parameter	H_3_O^+^		OH^−^	
	Induction	Progressive conversion	Induction	Progressive conversion
*E*_*A*_/J mol^−1^	109,400	68,300	82,700	75,700
*n*	0.47^50 °C^	1.2	1.1	1.1
	1.05^30 °C^			
$\frac{{bk_{0} }}{{\rho_{j} r_{0} }}$	8.07 × 10^16^	2.73 × 10^9^	7.30 × 10^16^	8.97 × 10^14^

## Discussion

### Dissolution experiments and stoichiometry

It was found that in all of the dissolution experiments, the dissolution reaction is extremely dependent on temperature and pH. The highest dissolution rates were obtained at high temperatures and high [H_3_O^+^]/[OH^−^] concentrations (see Tables 2, 3). For instance, in the dissolution in OH^−^medium at a pH of 12.29 and a temperature of 60 °C (343 K), the steady state was reached just after 2 min of reaction. On the other hand, the slowest reaction in this medium was obtained in the experiment with pH 10.79 and T = 30 °C (303 K), and the steady state was reached after 2100 min (≈1.5 days). The reactions in H_3_O^+^ were slower compared to those conducted in OH^−^medium, with the quickest reaction at a pH of 0.21 and a temperature of 80 °C (353 K), reaching the steady state after 8 min of reaction, and the slowest at pH 1.69 and T = 30 °C (303 K), where the steady state was reached after 13,500 min (≈10 days). All the conducted reactions underwent a pH change during the reaction, and it was more evident in the reactions in OH^−^ medium. These changes in pH are related to the high consumption of OH^−^/H_3_O^+^ions, mostly at the start of the reaction, corresponding to short induction periods. This fact is related to the reaction orders (*n*) that correspond to the induction period in both reaction media (Table 4). It can be noticed that the reaction order in OH^−^medium (*n* = 1.1) is slightly higher than that observed in H_3_O^+^ medium (*n* = 1.05 and 0.47). This difference is related to a higher dependence of the reaction on [OH^−^]; therefore, there is a higher OH^−^consumption, which is reflected in a drastic pH decrease.

Likewise, all the reactions showed a constant increase in K^+^, SO_4_^2−^ and CrO_4_^2−^concentrations during the reaction progress until the steady state was reached and concentrations became constant. As it can be noticed on Fig. 6a, the release rate of K^+^, SO_4_^2−^ and CrO_4_^2−^ into the solution is almost the same. Elwood Madden et al. [31] suggest that the dissolution rates are controlled by the bond breakage on *Y*-site, which in this case is the Fe–O bond, and not by the bond breakage on sites *M*, *Z* or OH^−^/H_2_O. Results of the dissolution experiments in H_3_O^+^ medium proved to be congruent, since in most of the cases, a complete dissolution of the solids was reached, and the K^+^, Fe^3+^, SO_4_^2−^ and CrO_4_^2−^ concentration in the remaining solution is stoichiometric according to the initial solid amount. Besides, as the XRD results in Fig. 7b show it, there was no formation of new phases during the dissolution reaction. In cases where solid dissolution was not completed, steady state was considered for the calculus of X. Thus, under the conditions used for this study, the dissolution of KFe_3_[(SO_4_)_2 − X_(CrO_4_)_X_](OH)_6_ in acidic medium can be described by the following general reaction:

7 $$\begin{aligned}
&\left[ {{\text{K}}_{1 - x} \left( {{\text{H}}_{ 3} {\text{O}}}
\right)_{x} } \right]{\text{Fe}}_{y} \left[ {\left( {{\text{SO}}_{
4} } \right)_{ 2- z} \left( {{\text{CrO}}_{ 4} } \right)_{z} }
\right]\left[ {\left( {\text{OH}} \right)_{w} \left( {{\text{H}}_{
2} {\text{O}}} \right)v} \right]_{{({\text{sol}})}} + (w -
x){\text{H}}_{ 3} {\text{O}}^{ + }_{{({\text{aq}})}} \hfill
\\
&\quad \to \left( {1 - x} \right){\text{K}}^{ + }_{{\left(
{\text{aq}} \right)}} + y{\text{Fe}}^{ 3+ }_{{\left( {\text{aq}}
\right)}} + (2 - z){\text{SO}_{ 4({\text{aq}})}^{ 2-}} +
z{\text{CrO}_{4({\text{aq}})}^{ 2-}} + (2w + v){\text{H}}_{ 2}
{\text{O}}_{{({\text{liq}})}} \end{aligned}$$

**Fig. 11:**
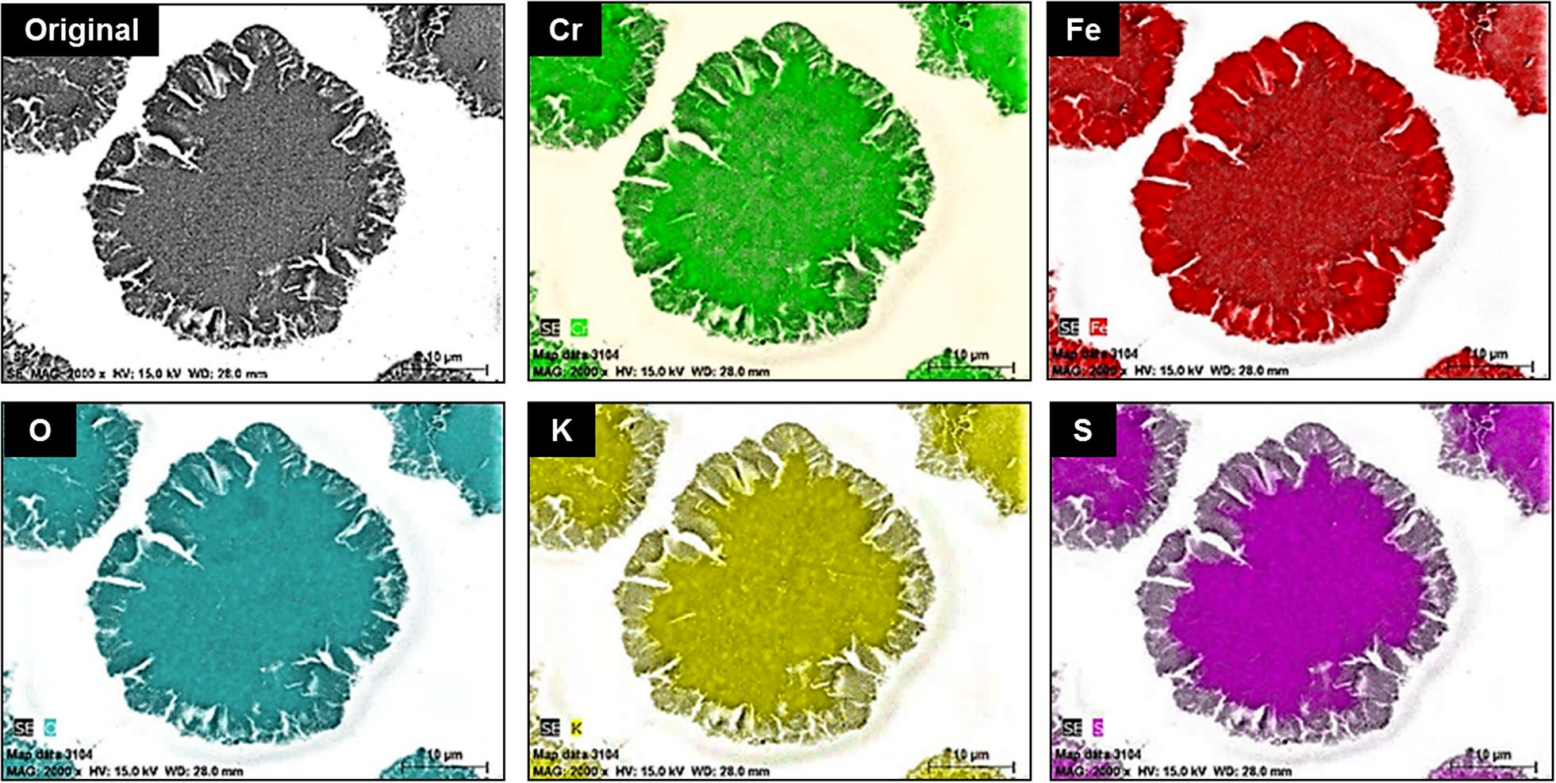
Energy dispersive X-ray mapping of a partially decomposed KFe_3_[(SO_4_)_2 − X_(CrO_4_)_X_](OH)_6_ particle in NaOH medium; pH 12.14, T = 30 °C (303 K), d_0_ = 38–44 µm, RPM = 750 min^−1^. The results confirm the unreacted core model and the adsorption of CrO_4_^2−^on the Fe(OH)_3_ halo

The reactions in OH^−^ were incongruent. This is indicated by the solid residues found at the end of each reaction. Several reaction products in the determination of solubilities and reaction rates of jarosite-type compounds have been proposed, although the complete identification of these phases is not yet convincing. Phases such as iron hydroxide, iron oxyhydroxide, ferrihydrite, schwermannite, goethite, hematite, lepidocrocite and maghemite have been suggested [31, 27, 33, 51–57]. The solid residues were identified by FT-IR and XRD analysis as amorphous Fe(OH)_3_ (Fig. 7a). On the other hand, the concentration of K^+^ and SO_4_^2−^ in the remaining solution proved to be very similar to that of the initial solids; instead, the concentration of CrO_4_^2−^ proved to be non-stoichiometric, as the concentration of CrO_4_^2−^ in the solution was slightly different from that of the initial solids at the end of the dissolution reaction. This inconsistency in the molar proportion of CrO_4_^2−^ between the solid residues and the remaining solution suggests that a small portion of the released CrO_4_^2−^ is adsorbed by Fe(OH)_3_ during the dissolution reaction. These results can be verified with a mapping of the different elements that form KFe_3_[(SO4)_2 − X_(CrO_4_)X](OH)_6_ in a partially decomposed particle. Figure 11 shows how Cr, Fe and O are present throughout the particle, while K and S can only be seen in the core, which indicates that they have diffused into the solution. Richards and Bourgs [58] mentioned that CrO_4_^2−^ ions can be adsorbed by Mn, Al, and Fe oxides; clay minerals and natural soils and colloids; and this adsorption is strongly dependent of pH. At dilute concentrations, adsorption of CrO_4_^2−^ increases as pH decreases whatever the adsorbent; also the adsorption is favored on adsorbents which are positively charged at low to neutral pH, i.e. which have high pH–ZPC values. Zachara et al. [59] reported that in alkaline environments, sorption is not strong enough to keep CrO_4_^2−^ over a solid surface (i.e. amorphous iron oxyhydroxide), but competitive adsorption with cations have a little influence on CrO_4_^2−^ adsorption. The pH adsorption edge is slightly shifted to higher alkaline pH due the presence of mayor cations such as K^+^—that is the case for this study-, Ca^2+^ and Mg^2+^. Cation adsorption enhances the positive charge and favors electrostatic adsorption of anions such as CrO_4_^2−^. As can be seen in Fig. 13, there is an almost imperceptible adsorption of K in the ash, that probably favor the slightly adsorption of CrO_4_^2−^. On the other hand, competing anions have a drastic effect. The effect will vary, depending on dissolved concentrations of the competing anion and CrO_4_^2−^. A shift of the pH adsorption edge towards lower pH values was generally observed, i.e. SO_4_^2−^, H_2_SiO_4_^2−^, among others. For the case of the dissolution reactions of KFe_3_[(SO_4_)_2 − X_(CrO_4_)X](OH)_6_, concentrations of SO_4_^2−^ and CrO_4_^2−^ always were similar in the solution (see formulas in Table 1), since both anions presented similar dissolution rates (see Fig. 6) and in some cases the CrO_4_^2−^ concentration was higher, probably this limits the competition between both anions, being favored the adsorption of chromate. Therefore, the dissolution reaction of KFe_3_[(SO_4_)_2 − X_(CrO_4_)_X_](OH)_6_ in alkaline medium can be described by the following general reaction (under the conditions used for this study):

8 $$ \begin{aligned}
&\left[ {{\text{K}}_{1 - x} \left( {{\text{H}}_{ 3} {\text{O}}}
\right)_{x} } \right]{\text{Fe}}_{y} \left[ {\left( {{\text{SO}}_{
4} } \right)_{ 2- z} \left( {{\text{CrO}}_{ 4} } \right)_{z} }
\right]\left[ {\left( {\text{OH}} \right)_{w} \left( {{\text{H}}_{
2} {\text{O}}} \right)_{v} } \right]_{{({\text{sol}})}} + (3y + x -
w){\text{OH}}^{ - }_{{({\text{aq}})}} \\ &\quad \to \left( {1 - x}
\right){\text{K}}^{ + }_{{\left( {\text{aq}} \right)}} +
y{\text{Fe}}\left( {\text{OH}} \right)_{ 3} \cdot t{\text{CrO}}_{
4({\text{sol}})}^{ 2- }+ u{\text{CrO}}_{ 4( {\text{aq}})}^{ 2-} + (2
- z){\text{SO}}_{ 4({\text{aq}})}^{ 2-} + (2x + v){\text{H}}_{ 2}
{\text{O}}_{{({\text{liq}})}} , \\
&\qquad\qquad t + u = z\\ \end{aligned}$$

SEM–EDS results (Figs. 4, 5), as well as the results shown on Fig. 8, confirm that the unreacted core shrinking model satisfactorily describes the dissolution process of KFe_3_[(SO_4_)_2 − X_(CrO_4_)_X_](OH)_6_. Dissolutions in OH^−^ medium are characterized by the formation of a halo of solid residues made of Fe(OH)_3_ that surrounds an unreacted core, while K^+^ and SO_4_^2−^ are preferentially released from the particle into the solution. The presence of this layer does not limit the dissolution rate, because, according to the calculations shown in Fig. 8, the stage that controls the process, or slow stage, is the chemical reaction (breakage of Fe–O bonds) on the surface of the unreacted core. Similarly, the stirring rate has no effect on the dissolution rates, because being a chemical control, the reagent diffusion process is quicker compared to the chemical reaction, even if at low [H_3_O^+^], diffusion plays an important role. In the same manner, according to SEM–EDS results (Fig. 5), the decompositions in H_3_O^+^ medium at low [H_3_O^+^] are described by the unreacted core model without the formation of the solid product layer, even if the results in Fig. 8 point to an intermediate behavior between controlling stages (diffusion in the ash layer and diffusion in the fluid membrane). The conversion-time equations (Eqs. 2–4) consider a single resistance throughout the reaction of the particle. However, the relative importance of diffusion of the fluid film in both the ash layer and the reaction stage varies as the reaction progresses. For a particle of constant size, the resistance in the fluid film remains constant and the resistance of the reaction diminishes, as the surface of the particle unreacted core decreases. The resistance of the ash layer does not exist at the beginning of the reaction (since there is not ash); but it becomes progressively more important as the ash layer is formed. Perhaps, it is not reasonable to consider that a unique stage controls the rate of the overall reaction. On the other hand, when a solid ash is formed during the reaction (as in the case of the dissolution reactions in OH^−^ media), the resistance of this layer is much greater than the resistance through of the fluid film that surrounds the particle. Therefore, the resistance of the fluid film may be neglected when the reaction does not form a not-flaky ash. In addition, the resistance of the ash layer is not affected by changes in the fluid velocity that surrounds the particle. In the case of reactions carried out at low concentrations of H_3_O^+^, when the controlling stage was the matter transport in the ash layer (although SEM results did not show formation of such layer at the reaction conditions used in this study), it is possible that the solution being strongly stirred (750 min^−1^) eliminates the resistance of the fluid layer, staying only the resistance offered by the flaky ash, as it is shown in Fig. 5b. The presented results are fairly coherent with previous studies on the dissolution of jarosite-type compounds [30, 39, 40–45, 51].

**Fig. 12:**
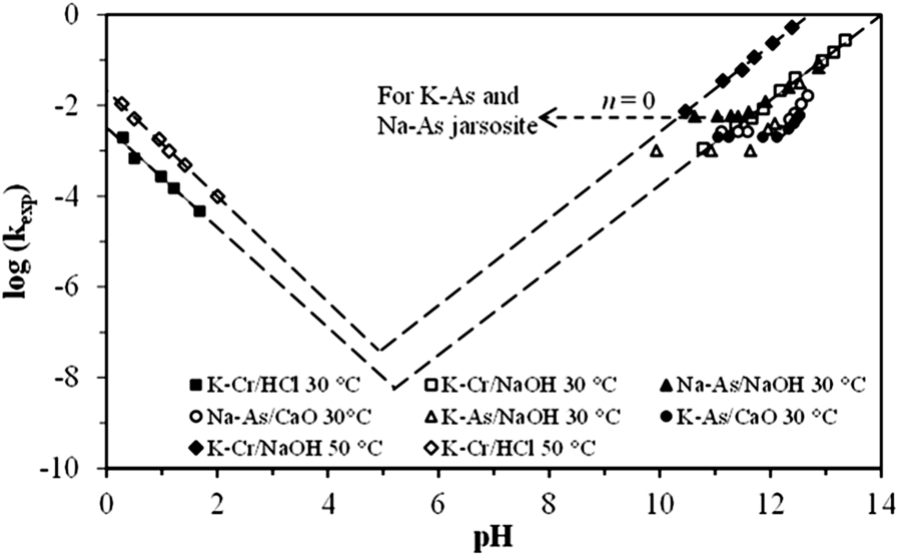
Dissolution rate constants (k_exp_) vs. pH of the reaction, and total reaction times (t_r_) for the dissolution of KFe_3_[(SO_4_)_2 − X_(CrO_4_)_X_](OH)_6_. Results show that the dissolution rate increases as the ratio OH^−^/H_3_O^+^ is increased in the system. Values of rate constants and t_r_ for the dissolutions of K-As-jarosite and Na-As-jarosite at 30 °C are very similar to the ones reported in this work. Figure 12 illustrates data taken from: ^a^this work, ^b^Patiño et al. [32, 33] and ^c^Flores et al. [51]

The dissolution rates obtained in this study are similar to rates previously obtained in other studies on synthetic jarosite-type compounds: NH_4_, Ag, K-As, Na-As, Pb-Ag, Ag-NH_4_, Na-Ag. As it can be noticed, the substitutions are site *M* and site *Z*; it is worth mentioning that these studies were conducted in the initial stage of the reaction (far from equilibrium) in OH^−^ medium [30, 39, 40–45, 51]. For instance, in Fig. 12, the results of the dissolution of K-As and Na-As jarosites in NaOH and CaO media are compared to the results obtained for this paper. It can be seen that the behavior of the dissolution rates is very similar, even if they were conducted at different [OH^−^] and T conditions. This indicates that the substitutions, whether on *Y*-site or Z-site, have little effect on the dissolution rates of jarosite-type compounds. In addition, under alkaline conditions, the dissolution produces secondary solids (iron hydroxide). However, Flores et al. [51] and Patiño et al. [33] found that, for the dissolution of K-As and Na-As jarosites in NaOH and CaO media, at a pH of ≈11.5 or lower, there is a reaction order of *n* = 0 (Fig. 12), which suggests that there is no dependence of the dissolution reaction on the [OH^−^] of the medium. Kendall et al. [34] suggest that the incorporation of As in the jarosite structure limits the efficiency of the OH^−^attack on the particle surface, resulting mostly in a H_2_O attack. This change in the mechanism can be due to the arsenate bonds on the surface, creating electrostatic repulsion of the hydroxyls at a high pH; besides, the increase in the number of Fe-AsO_4_ bonds inhibits these systems’ dependence on [OH^−^]. This phenomenon was not observed in the dissolution of K-Cr-jarosites since they were not found reaction orders n = 0, indicating a continuous dependency of the reactions towards concentrations of H_3_O^+^/OH^−^.

### Effect of [H_3_O^+^]/[OH^−^], T, d_0_ and SO_4_^2−^/CrO_4_^2−^proportion in the structure on the dissolution rate of KFe_3_[(SO_4_)_2 − X_(CrO_4_)_X_](OH)_6_

The dissolution of KFe_3_[(SO_4_)_2 − X_(CrO_4_)_X_](OH)_6_ shows a high dependence on [H_3_O^+^] and [OH^−^]; the calculated reaction orders (Table 4) show that the dependence of the dissolution reaction on the reaction medium is similar for both reaction media and periods, because the calculated reaction order is n = 1.0 for all the cases. This *n* value indicates that the obtained dissolution rates are directly proportional to the reactant concentration, which means that low concentrations correspond to low reaction rates, and vice versa. Nonetheless, the reaction order calculated for the induction period in [H_3_O^+^] medium at 50 °C, proved to be much lower than expected (*n* = 0.47), which indicates that [H_3_O^+^] concentration under these conditions has little influence in the reaction, even at high concentrations, as the beginning of the reaction is mainly affected by temperature.

Temperature was the variable with the strongest effect on the dissolution of KFe_3_[(SO_4_)_2 − X_(CrO_4_)_X_](OH)_6_. For instance, for one same [H_3_O^+^], at 30 °C (303 K), the reaction had a duration of 2280 min, while at 80 °C (353 K), the reaction reached the steady state in only 30 min. In the same way, temperature affects the dissolution rate in the induction period to such extent, that at high reaction temperatures, this period disappears. Therefore, the formation of active sites and the beginning of the progressive conversion period are instantaneous. The energy dependence (*E*_*A*_) calculated in the progressive conversion period was lower than in the induction period in both reaction media (see Table 4). This noticeable difference is related to the difficulty in chemical adsorption and subsequent establishment of a reaction front of H_3_O^+^/OH^−^ ions on the superficial active centers, which are very stable, so the energy demand is higher in the induction period.

**Fig. 13:**
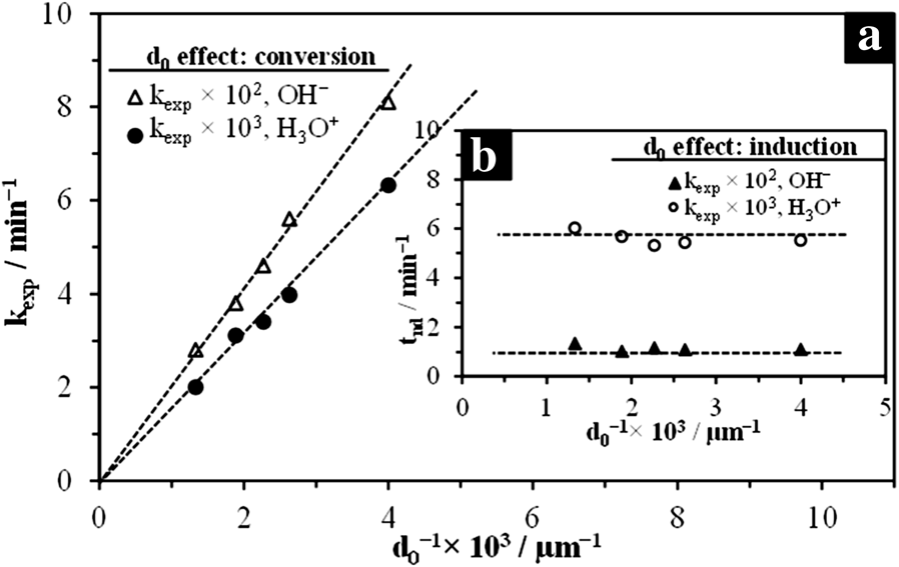
Plot of the reaction rate constants (k_exp_) vs. the inverse of the particle’s initial diameter for both reaction media. **a** Progressive conversion period; **b** induction period. The results confirm that the unreacted core model satisfactorily describes the dissolution of KFe_3_[(SO_4_)_2 − X_(CrO_4_)_X_](OH)_6_

According to Eq. 5, a representation of the experimental constants determined at constant temperature and concentration vs. the inverse of the particle diameter, should be linear and pass through the origin. Figure 13a presents the dependence of k_exp_ on the particle’s initial diameter d_0_. From this plot we can deduce that the experimental constant is inversely proportional to the particle diameter (k_exp_ α 1/d_0_), so the dissolution of KFe_3_[(SO_4_)_2 − X_(CrO_4_)_X_](OH)_6_ in H_3_O^+^/OH^−^ medium is consistent with the unreacted core model with chemical control. However, the induction period is practically independent from the particle size, as observed in Fig. 13b.

**Fig. 14:**
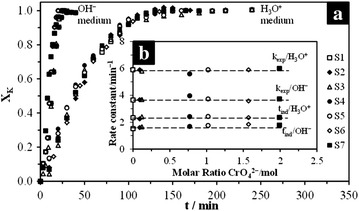
Dissolution curves for S_1_–S_7_; **a** [H_3_O^+^]/[OH^−^] = 0.1 mol L^−1^, T = 50 and 30 °C respectively, d_0_ = 44–38 µm. **b** Comparison of the calculated rate constants vs. CrO_4_^2−^ proportion in the structure of potassium jarosite. Results show that the incorporation of the chromate ion does not modify the dissolution rate

Results of the experiments on the dissolution of potassium jarosite, its chromate analog and the 5 synthesized solid solutions (S_1_–S_7_), conducted at constant [H_3_O^+^]/[OH^−^], T and d_0_, are shown in Fig. 14. As it can be seen in the dissolution curves (Fig. 14a), similar dissolution rates were found in the 7 dissolution experiments, so the incorporation of CrO_4_^2−^ into the structure of potassium jarosite does not modify the dissolution rate, even when the substitution of CrO_4_^2−^ is total in both reaction media. The comparison of the calculated rate constants vs. SO_4_^2−^/CrO_4_^2−^proportion (Fig. 14b) clearly confirms that the dissolution rate is not modified and there is no behavior tendency due to the presence of the chromate ion in the structure. Likewise, these results can be applied to the substitution in the *M*-site, since, according to Table 1, several proportions of H_3_O^+^ in the structure are incorporated into the produced syntheses. This incorporation difference is more evident between S_1_ and S_7_. Results show that even a high substitution of H_3_O^+^ does not modify the dissolution rate. As previously mentioned, this similarity between dissolution rates is due to the fact that the stage limiting the reaction is the chemical reaction with the breakage of Fe–O bonds on the surface of the particle. Although the nature of the species that substitutes in *Z*-site can have an influence on the dissolution rates (for example, on the dissolution rates of K-As and Na-As jarosite), it was found that the dissolution rate is modified when the incorporation of As is increased.

### Implications

The results of this and other studies indicate that jarosite-type compounds are excellent deposits for elements of environmental importance, such as Cr(VI) and As(V), because they have high stability in a wide range of pH and T. They are more stable at an acidic pH and low T, e.g., according to the results obtained in this study, K-Cr jarosite showed extremely slow dissolution rates at a pH 1.5, indicating that a high pH value will result in even slower dissolution rates. On the other hand, even though the stability in alkaline media was lower compared to the dissolutions in acidic medium and relative to quicker dissolution rates even at moderately high pH values (pH 10.5), the release of Cr(VI) into the solution is not immediate, because after the dissolution of KFe_3_[(SO_4_)_2 − X_(CrO_4_)_X_](OH)_6_, there is a quick adsorption of Cr(VI) on the residues made of Fe(OH)_3_, which delays this element’s access into the environment. Therefore, the precipitation of phases such as KFe_3_[(SO_4_)_2 − X_(CrO_4_)_X_](OH)_6_, and the adsorption of Cr(VI) after the dissolution can play an important role as retention mechanisms of Cr(VI) in nature. In addition, chromate ions can also be adsorbed by aluminum oxides, kaolinite, montmorillonite, organic complexes and other clay minerals that are common components of soil. This adsorption is favored by a decrease in pH, and it was found that the highest chromate adsorption is in acidic to neutral conditions in the presence of iron oxyhydroxides [60] such as hematite, schwertmannite, maghemite and ferrihydrite; that are products of the dissolution reaction of the jarosite-type compounds and presence of any of these iron oxyhydroxides vary according as function of temperature and pH [31]. Moreover, the fact that sulfate is preferentially incorporated into KFe_3_[(SO_4_)_2 − X_(CrO_4_)_X_](OH)_6_ formed by synthetic acid solutions implies that the formation of this phase is possible even at low sulfate concentrations, and the fact that the yield of the precipitation reaction increases along with the sulfate concentration indicates that the capability of KFe_3_[(SO_4_)_2 − X_(CrO_4_)_X_](OH)_6_ to keep low Cr(VI) concentrations in solution is better than a pure phase of KFe_3_(CrO_4_)_2_(OH)_6_. Thus, KFe_3_[(SO_4_)_2 − X_(CrO_4_)_X_](OH)_6_ could limit the mobility of Cr(VI) more than the precipitation of KFe_3_(CrO_4_)_2_(OH)_6_ [4]. It was also found that the incorporation of CrO_4_^2−^ and H_3_O^+^ does not modify the dissolution rates, which suggests that regardless of the SO_4_^2−^/CrO_4_^2−^ or K^+^/H_3_O^+^ proportion in the structure, the dissolution process is the same.

The dissolution of KFe_3_[(SO_4_)_2 − X_(CrO_4_)_X_](OH)_6_ showed a directly proportional dependence on [H_3_O^+^]/[OH^−^], which is represented by the reaction orders calculated for both media and periods. A value of *n* = 1 (Table 2) indicates that the reaction rate is directly proportional to [H_3_O^+^]/[OH^−^]: very high concentrations are necessary for quick reaction rates, and vice versa. Consequently, a low [H_3_O^+^]/[OH^−^] concentration will cause the beginning of the reaction to be slow, thus delaying the incorporation of Cr(VI) into the environment. An important piece of data obtained in this study is the pH value at which the dissolution of KFe_3_[(SO_4_)_2 − X_(CrO_4_)_X_](OH)_6_ is instantaneous, without an induction period calculated from the representation of log (1/tind) vs. log [H_3_O^+^] or log [OH^−^] (Fig. 9) and the intersection of the linear regression with the x axis (log [H_3_O^+^] or log [OH^−^]). For the dissolutions in acidic medium, this value was pH 0.05, and in alkaline medium it was pH 13.4 at 30 °C, which indicates that extremely acidic or alkaline conditions are necessary for an instant dissolution. Patiño et al. [33] obtained a similar value for the dissolution of Na-As jarosite in alkaline medium (pH 13.6). The energy dependence calculated for the progressive conversion period in both media was much lower (almost half the value) than the *E*_*A*_ in the induction period. Consequently, low temperatures will result in slow dissolution rates, even at high [H_3_O^+^]/[OH^−^] (see Tables 2, 3). It was also found that the reaction rates decrease as the initial particle diameter grows, so larger initial particle diameters will facilitate slower dissolution rates, although the particle size effect was not as strong as the effect of T and [H_3_O^+^]/[OH^−^] on the obtained dissolution rates.

## Conclusions

Potassium jarosite, its chromate analog and 5 solid solutions with different SO_4_^2−^/CrO_4_^2−^ proportions in the structure were synthesized. The effect of T, [H_3_O^+^]/[OH^−^], d_0_, SO_4_^2−^/CrO_4_^2−.^on the dissolution rate of these phases was studied. The experimental results indicate that the reaction rate is highly dependent on temperature, closely followed by pH of the reaction solution. Generally speaking, the order of importance regarding the effect of the studied variables is as follows: T > [H_3_O^+^]/[OH^−^] > d_0_ > SO_4_^2−^/CrO_4_^2−^. It was also found that the incorporation of Cr(VI) in the structure does not affect the dissolution rate. Extreme pH conditions (acidic or alkaline) cause the preferential release of K^+^, SO_4_^2−^ and CrO_4_^2−^ from the KFe_3_[(SO_4_)_2 − X_(CrO_4_)_X_](OH)_6_ structure, although CrO_4_^2−^ is quickly adsorbed by Fe(OH)_3_ solid residues. Likewise, the experimental results are fairly consistent with the unreacted core kinetic model with formation of a solid sub product layer. In most of the reactions, the chemical reaction is the stage controlling the dissolution process, although in the reactions at low [H_3_O^+^] (1.5 ≥ pH ≤ 4.5) and T ≤ 30 °C, the diffusion of H_3_O^+^ ions on the unreacted core can play an important role in the dissolution rate of KFe_3_[(SO_4_)_2 − X_(CrO_4_)_X_](OH)_6_. The kinetic analysis related to the reaction orders and calculated activation energies confirmed that extreme pH and T conditions are necessary to obtain considerably high dissolution rates. Therefore, inside the common pH and T intervals of water bodies and superficial soils, it can be considered that the precipitation of KFe_3_[(SO_4_)_2 − X_(CrO_4_)_X_](OH)_6_ can work as Cr(VI) deposit and thus limit its environmental mobility, since it offers a high stability in acidic media. Similarly, the quick adsorption of Cr(VI) on iron residues after the dissolution of KFe_3_[(SO_4_)_2 − X_(CrO_4_)_X_](OH)_6_, offers an additional deposit in environments with neutral to slightly alkaline pH, which is an unfavorable condition for jarosite-type compounds. To make a proper comparison with other dissolution rate values, it is necessary to establish the validity of the kinetic model used in this work mainly for intermediate pH values (2–10), which are the most common conditions found in nature. According to the results, there are differences between the dissolution rates observed under extreme conditions of pH and those obtained at intermediate conditions. Especially in the controlling stage of the dissolution rates, being dominant the chemical reaction in the dissolution reactions at extreme conditions of pH (2.0 ≤ pH ≥ 10.0); and mass transport in the residual solid layer in the reactions at intermediate pH conditions. It is also necessary to consider that in reactions with intermediate pH, unreacted solid remains, calculations of the reaction rates are made in the steady state, and it is not considered the saturation condition of the system. For these reasons, it is still necessary to perform additional experiments taking in account these considerations.
